# Histopathological Account of Obstetrical and Gynecological Specimens: Retrospective Study at a Tertiary Care Center of Peshawar

**DOI:** 10.7759/cureus.14950

**Published:** 2021-05-11

**Authors:** Omer Nasim, Muhammad Khizar Hayat, Zeinab Hussain, Muhammad Shah Fahad, Ayesha Jamal, Mohammad Ahmed Arsalan Khan

**Affiliations:** 1 Trauma and Orthopaedics, Poole General Hospital, National Health Service, Poole, GBR; 2 General Surgery, Rehman Medical Institute, Peshawar, PAK; 3 Medicine, Rehman Medical College, Peshawar, PAK; 4 General Surgery, Combined Military Hospital, Peshawar, PAK; 5 Cardiothoracic Surgery, Rehman Medical Institute, Peshawar, PAK

**Keywords:** uterus, histopathology, ovary, gynaecology and obstetrics, cervix

## Abstract

Introduction

Histopathologic specimen examination of surgically isolated organs and tissues yields valuable information regarding a disease process and plays a vital role in the future management of a patient. Our aim was to account for the common diagnosis yielded from histopathological specimens of the obstetrics and gynecology department and to determine if all the obstetric and gynecological specimens should be routinely sent for histopathology.

Methods

A retrospective, cross-sectional study was conducted at the histopathology unit of a tertiary care hospital in Peshawar. Data were acquired for all gynecological and obstetric specimens sent for histopathology for analysis to the histopathology unit during August 2018 and July 2019. Any sample that was not sent via surgical excision was excluded from the study.

Results

A total of 922 samples were sent for histopathological analysis in the tertiary care hospital. The mean age of patients who had their specimens sent for pathology was 40.78 ± 10.81 years. Most of the samples sent were of the uterus (458) and the age 31-50 years (270) had the highest proportion of histopathological specimens. Normal ovaries (64.4%) and fallopian tubes (78.8%) were the main diagnoses for these two specimens while a normal cervix (0.58%) was the least common diagnosis among samples sent for histopathology. Chronic cervicitis (92.4%) in cervix and secretory phase endometrium (30.1%) in the uterus were the other common diagnosis. All the other samples were infrequently sent.

Conclusion

Uterine specimens are the most common histopathological specimen sent followed by cervix and then fallopian tube. Fallopian tube and ovaries yielded the highest normal diagnosis. Cervix specimens must be biopsied. More data is needed for a certain consensus on the need for routine histopathology.

## Introduction

Histopathologic specimen examination of surgically isolated organs and tissues plays a crucial role in the confirmation of a diagnosis. Most surgical specimens need to be submitted for further histopathological analysis, as it may yield valuable information relevant for the future management of the patient [1]. The College of American Pathologists has presented specific guidelines for specimen collection and submission and has given an account of the specimens that can be excluded from being sent for histopathology [2]. Though the final decision on specimen submission lies with the governing body of an institution. It is suggested that it should be scrutinized in a multidisciplinary team meeting for suitability and only then be sent for histopathological examination.

Histopathological examination of surgical specimens has varied significance, it could be therapeutic or diagnostic, legal or ethical [3]. The diagnostic value of histopathology has been well established in genital carcinomas and adenomyosis. It is also a useful tool for the diagnosis of exclusion in dysfunctional uterine bleeding. Hysterectomy is a commonly performed surgical procedure that aids in the sampling and diagnosis of various benign and malignant diseases of the female reproductive system [4]. Cervical cancer is another entity diagnosed with histopathology and it is the second most common gynecological malignancy worldwide and the third most common cause of mortality in women worldwide [5]. In Pakistan, however, according to different studies, Ovarian cancer is the most common gynecological malignancy [6].

Even though there is nationwide and international consensus on which types of specimens deserve pathologic examination and which do not, there are still discussions about the necessity of some pathologic examinations [1]. Pakistan being an underdeveloped country, not many people can afford surgeries, and then the added routine examination without an indication can be an extra burden for the patient and their family. This study aims to account for the common diagnosis yielded from histopathological specimens of the obstetrics and gynecology department of a tertiary care hospital and to determine if all obstetric and gynecological specimens should be routinely sent for histopathology.

## Materials and methods

A retrospective, cross-sectional study was conducted at the histopathology unit of a tertiary care hospital in Peshawar. Data were acquired for all gynecological and obstetric specimens sent to histopathology for analysis during August 2018 and July 2019. Any sample that was not sent via surgical excision was excluded from the study. Data collected included, patient record number, age, gender, specimen type, and diagnosis. The acquired data was in the form of a Microsoft Excel database, password-protected for confidentiality and only to be used for this study. The spreadsheet was analyzed for descriptive statistics and pivot tables were generated. Data were presented in the form of tables.

## Results

A total of 922 samples were sent for histopathological analysis to the tertiary care hospital histopathology unit. The mean age of patients who had their pathology sent was 40.78 ± 10.81 years.

Table 1 shows the age and organ division. It is clear that most of the samples sent were of the Uterus (458) predominantly of age 31-50 years (270). Labia, umbilicus, and vaginal specimens (2 each) were least commonly sent for histopathology. One ovarian sample of a four-year-old child was also sent which is not included in the table. Age 31-50 years (660) is the most common age for obstetric and gynecological specimens sent for histopathology.

**Table 1:** Age and organ division. *One ovary sample was from four-year-old.

Age groups* (years)	Cervix (n = 171)	Fallopian tube (n = 165)	Labia (n = 2)	Ovary (n = 187)	Placenta (n = 1)	Umbilicus (n = 2)	Uterus (n = 458)	Vagina (n = 2)	Vulva (n = 3)	Total
10-30	6	13	-	28	1	-	139	1	2	190
31-50	132	123	2	131	-	-	270	1	1	660
51-70	32	28	-	28	-	-	44	-	-	134
71+	1	1	-	-	-	0	5	-	-	7

Table 2 shows the histopathological diagnosis along with the surgical specimen sent. Normal ovaries (64.4%) and fallopian tubes (78.8%) were the main diagnoses for these two specimens while a normal cervix (0.58%) was the least common diagnosis among samples sent for histopathology. Mature cystic teratoma (6.9%) and endometriotic cysts (6.9%) were the second most common ovarian finding while Unilateral benign paratubal cysts (12.1%) were the second common diagnosis in fallopian tube specimens. Chronic cervicitis (92.4%) in the cervix and secretory phase endometrium (30.1%) in the uterus was the other common diagnosis. All the other samples were infrequently sent.

**Table 2:** Organs with their diagnosis. Ca: Carcinoma.

Organ	Diagnosis	n (%)
Ovary (188)	Normal	121 (64.4)
	Mature cystic teratoma	13 (6.9)
	Ectopic pregnancy	2 (1.1)
	Serous cystadenoma	10 (5.3)
	Mucinous cystadenoma	5 (2.7)
	Endometriotic cysts	13 (6.9)
	Ovarian torsion	3 (1.6)
	Follicular cysts	8 (4.3)
	Luteal cyst	9 (4.8)
	Granulosa cell tumor	2 (1.1)
Fallopian tube (165)	Normal	130 (78.8)
	Unilateral benign paratubal cysts	20 (12.1)
	Bilateral benign paratubal cysts	5 (3.0)
	Ectopic pregnancy	7 (4.2)
	Adenomatous polyp	1 (0.6)
	Acute on chronic salpingitis	1 (0.6)
Cervix (171)	Chronic cervicitis	158 (92.4)
	Nabothian cyst	127 (74.3)
	Cervical polyp (benign)	18 (10.5)
	Normal	1 (0.6)
	Squamous metaplasia	45 (26.3)
	Adenocarcinoma	1 (0.6)
	Squamous cell Ca	2 (1.2)
	Hyperkeratosis	3 (1.8)
Uterus (458)	Leiomyoma	111 (24.2)
	Adenomyoma	86 (18.8)
	Chronic endometriosis	12 (2.6)
	Atrophic endometrium	18 (3.9)
	Benign endometrial polyp	19 (4.1)
	Hydatidiform mole	5 (1.1)
	Partial mole	1 (0.2)
	Normal	2 (0.4)
	Metastatic Ca	1 (0.2)
	Products of conception	90 (19.6)
	Proliferative phase endometrium	138 (30.1)
	Secretory phase endometrium	87 (19.0)
	Papillary adenocarcinoma	1 (0.2)
	Endometroid carcinoma	2 (0.4)
	Inactive endometrium	4 (0.9)
	Decidual tissue only	20 (4.4)
Labia (2)	Benign cyst	1 (50.0)
	Fibromatosis	1 (50.0)
Placenta (1)	Products of conception	1 (100)
Umbilicus (2)	Mild chronic non-specific inflammation	1 (50.0)
	Adenocarcinoma with neuroendocrine features	1 (50.0)
Vagina (2)	Bartholin cyst	1 (50.0)
	Metastatic adenocarcinoma	1 (50.0)
Vulva (3)	Condyloma acuminata	1 (33.3)
	Lichen simplex chronicus	1 (33.3)
	Verica vulgaris	1 (33.3)

## Discussion

The majority of the samples sent for histopathology in our study were from the age group of 31-50 years (71.6%). This range is quite typical for gynecological problems that result in sampling, particularly around the late forties, as observed in several of the other studies referenced [3,4,7,8]. This is probably due to this being the peak reproductive age and females are more likely to present to a primary physician as compared to other age groups.

In our study, the most common pathological diagnosis for uterine samples was leiomyoma. Out of 458 uterine samples in our study 24.2% were diagnosed as leiomyoma, followed by 18.8% being adenomyoma. These findings are mirrored by studies conducted in Lahore and Karachi, the highest proportion of diagnosis being leiomyoma in uterine samples, 69% and 66%, respectively [4,7]. The latter also had adenomyosis as the second most common finding at 21%. Uterine leiomyomas, also called fibroids, and are the most common pelvic tumors in women [9]. On the other hand, a study in Nowshera showed adenomyosis to be the most common finding (49.8%), which was followed by leiomyoma (15.3%) [10]. 

The majority of ovarian findings in our study were normal (64.4%), followed in equal frequency by mature cystic teratomas and endometriotic cysts (6.9% each), and then serous cystadenomas (5.3%). Luteal cysts, a common finding in other studies, were only present in 4.9% of the ovarian samples in our setting. In a study by Seema Butt, et al. the most common lesions found in ovaries were follicular cysts and corpus luteal cysts [11]. A 10-year research showed corpus luteal cysts and serous cystadenomas as the most common findings on histopathology of the ovaries [12]. Another study on ovarian tumors showed serous cystadenomas and mature cystic teratomas as the most common surface epithelial and germ cell tumors respectively [13]. A significant trend could not be observed from the present literature hence, more research would have to be done to confirm any consistency of the frequency of ovarian diseases.

Chronic cervicitis was present in 92.4% of the cervical samples in our setting. It is found to be the most common pathology reported on histopathology of the cervix in several studies [3,4,7]. Histopathology is therefore a vital investigation for the diagnosis of common cervical disease, including cervicitis and cervical cancer. Samples should be routinely sent for histopathology to confirm their diagnosis. The majority of the cervix specimens sent yielded a diagnosis which is another reason why cervix specimens must be sent for histopathological examinations. Hence, sending cervix specimens as routine can be made mandatory.

Unilateral benign paraovarian/paratubal cysts were the most frequent pathology diagnosed in fallopian tube samples (12.1%). Studies have shown that ultrasonography, particularly transvaginal ultrasounds, have an 87%-100% rate of accuracy in diagnosing these cysts, but histopathology of the specimen is required to differentiate between simple cysts, adenomatoid tumors, or cystadenomas [14,15] therefore, any of paraovarian or paratubal cysts should be sent for histopathology so that adenomatoid tumors or cystadenomas are not missed.

There was also a case of umbilical adenocarcinoma diagnosed in the samples of this study. It is a rare condition and has not been reported much in prevalence studies, but case studies have shown that histological support is needed to confirm the diagnosis. These tumors present as painful nodules of varying sizes and can also be ulcerated. These may also present as an abscess underlying the tumor [16,17]. Such a diagnosis found on routine sampling makes a case for all samples requiring histopathology so that no condition is left unnoticed and hence untreated. 

While histopathology is an important diagnostic tool, it is rather expensive for individuals of third-world countries like Pakistan where most of the patients do not have an insurance system to rely upon for their treatments. Sending products of conception for routine histopathology is a known dilemma, 19.6% of our center's uterine samples showed products of conception as a result. In a short communication published in the Australian and New Zealand Journal of Obstetrics and Gynaecology, it was stated that the commonest reason these samples are sent for histopathology is to avoid misdiagnosis [18]. This could be a genuine reason however, patients' financial status needs to be taken into account as well. Also, the doctor should have sufficient doubt to warrant a histopathological diagnosis else the only person that suffers is the patient.

One study found that routine endometrial sampling of asymptomatic women was unnecessary as it had a very low rate of detection of any pathologies [19]. Transvaginal ultrasounds are highly sensitive to leiomyomas and adenomyomas, the most recurring endometrial pathologies in our study, and hence the shift in paradigm might be considered [20]. On the other hand, biopsy and histopathological typing are found to be essential in better prognosis and management of ovarian tumors [13]. In one study, 74% of the pre-operative diagnoses were confirmed upon histopathology [3], highlighting the need for histopathology. A clear demarcation as to its need and benefit could not be established yet again.

A study by Akhter Shahida et al. between the pap smear, colposcopy, and cervical histopathology yielded that a strong agreement between the colposcopic findings and histopathological diagnosis existed however, an agreement between colposcopic and cytological findings and cytology and histopathological diagnosis was weak [21].

It is hard to lay out firm guidelines from the present data for mandating obstetric and gynecological specimens to be sent for routine histopathology. The decision lays in the hands of the surgeon. The surgeon needs to be partial to the patient’s socioeconomic status and evaluate the benefit before sending a routine for histopathology.

## Conclusions

Uterine specimens are the most common histopathological specimen sent followed by the cervix and then the fallopian tube. The fallopian tube and ovaries yielded the highest normal diagnosis. Cervix specimens must be biopsied as most reveal a positive diagnosis. Finally, more data is needed for a certain consensus on the need for routine histopathology.

## References

[1] Damjanov I, Vranic S, Skenderi F (2016). Does everything a surgeon takes out have to be seen by a pathologist? A review of the current pathology practice. Virchows Arch.

[2] Wilson ML (1996). General principles of specimen collection and transport. Clin Infect Dis.

[3] Naheed K, Hussain A, Ali R (2018). Clinico-pathological study of hysterectomy at Pak Red Crescent Medical and Dental College. J Islamic Int Med Coll.

[4] Rashid A, Qamar H, Pario S (2020). Frequency and morphology of benign histopathological lesions in total abdominal hysterectomy specimens. Professional Med J.

[5] Vinh-Hung V, Bourgain C, Vlastos G, Cserni G, De Ridder M, Storme G, Vlastos AT (2007). Prognostic value of histopathology and trends in cervical cancer: a SEER population study. BMC Cancer.

[6] Jamal S, Mamoon N, Mushtaq S, Luqman M, Moghal S (2006). The pattern of gynecological malignancies in 968 cases from Pakistan. Ann Saudi Med.

[7] Sarfraz R, Ahmed MM, Tahir TM, Ahmed MS (2011). Benign lesions in abdominal hysterectomies in women presenting with menorrhagia. Biomedica.

[8] Amin A, Ali A, Amin Z, Nighat Sani F (2013). Justification for hysterectomies and frequency of histopathological lesions of hysterectomy at a Teaching Hospital in Peshawar, Pakistan. Pak J Med Sci.

[9] Drayer SM, Catherino WH (2015). Prevalence, morbidity, and current medical management of uterine leiomyomas. Int J Gynaecol Obstet.

[10] Afridi N, Fareed A, Nazeer S, Khan S, Khan SG (2020). Histopathology diagnosis in women who underwent a hysterectomy for a benign condition. Professional Med J.

[11] Butt S, Fazili MH, Rathore S, Ibnerasa N (2019). Histopathological study to see the incidence of Neoplastic and Non-Neoplastic lesions in ovaries received in a Tertiary Care Hospital Lahore Pakistan. PJMHS.

[12] Izhar R, Zaheer N, Mansuri FA (2009). Histopathological profile of cases of oophorectomy: a report from 1997-2007. J Liaquat Univ Med Health Sci.

[13] Bashir SA, Waris EJ, Suleman BA, Qureshi GH (2011). Frequency of different histopathologic patterns of benign ovarian tumors in women with different age groups. PJMHS.

[14] Gupta A, Gupta P, Manaktala U, Khurana N (2016). Clinical, radiological, and histopathological analysis of paraovarian cysts. J Midlife Health.

[15] Savelli L, Ghi T, De Iaco P, Ceccaroni M, Venturoli S, Cacciatore B (2006). Paraovarian/paratubal cysts: comparison of transvaginal sonographic and pathological findings to establish diagnostic criteria. Ultrasound Obstet Gynecol.

[16] Dhull AK, Atri R, Kaur P, Singh G, Chauhan A (2010). Primary umbilical adenocarcinoma: a case report. Internet J Third World Med.

[17] Alnaqbi KA, Joshi S, Ghazal-Aswad S, Abu Zidan FM (2007). Primary umbilical adenocarcinoma. Singapore Med J.

[18] Yap SJ, Watts JC, Faithfull TJ, Wong SZ, Wylde KL, McGurgan PM (2014). Is tissue an issue? Current practice and opinion in Western Australia for routine histopathology on products of conception. Aust N Z J Obstet Gynaecol.

[19] Kapali M, Agaram NP, Dabbs D, Kanbour A, White S, Austin RM (2007). Routine endometrial sampling of asymptomatic premenopausal women shedding normal endometrial cells in Papanicolaou tests is not cost effective. Cancer.

[20] Hanafi M (2013). Ultrasound diagnosis of adenomyosis, leiomyoma, or combined with histopathological correlation. J Hum Reprod Sci.

[21] Akhter S, Bari A, Hayat Z (2015). Variability study between Pap smear, Colposcopy and Cervical Histopathology findings. J Pak Med Assoc.

